# Group II Introns Break New Boundaries: Presence in a Bilaterian's Genome

**DOI:** 10.1371/journal.pone.0001488

**Published:** 2008-01-23

**Authors:** Yvonne Vallès, Kenneth M. Halanych, Jeffrey L. Boore

**Affiliations:** 1 Department of Energy (DOE) Joint Genome Institute and Lawrence Berkeley National Laboratory, Walnut Creek, California, United States of America; 2 Department of Integrative Biology, University of California at Berkeley, Berkeley, California, United States of America; 3 Life Sciences Department, Auburn University, Auburn, Alabama, United States of America; 4 Genome Project Solution, Hercules, California, United States of America; University of California at Berkeley, United States of America

## Abstract

Group II introns are ribozymes, removing themselves from their primary transcripts, as well as mobile genetic elements, transposing via an RNA intermediate, and are thought to be the ancestors of spliceosomal introns. Although common in bacteria and most eukaryotic organelles, they have never been reported in any bilaterian animal genome, organellar or nuclear. Here we report the first group II intron found in the mitochondrial genome of a bilaterian worm. This location is especially surprising, since animal mitochondrial genomes are generally distinct from those of plants, fungi, and protists by being small and compact, and so are viewed as being highly streamlined, perhaps as a result of strong selective pressures for fast replication while establishing germ plasm during early development. This intron is found in the mtDNA of an annelid worm, (an undescribed species of *Nephtys*), where the complete sequence revealed a 1819 bp group II intron inside the *cox1* gene. We infer that this intron is the result of a recent horizontal gene transfer event from a viral or bacterial vector into the mitochondrial genome of *Nephtys* sp. Our findings hold implications for understanding mechanisms, constraints, and selective pressures that account for patterns of animal mitochondrial genome evolution

## Introduction

Ribozymes are RNA molecules with enzymatic activities [1]. One type is called a self-splicing intron because these are removed from the gene's transcript without the formation of the spliceosomal protein complex (although some may require other proteins for efficient splicing *in vivo*) [2]. These are separated into group I and II types depending on the mechanism of splicing [1], [3], [4]. Although each type folds into a characteristic structure that is necessary for catalysis, there is almost no widely conserved nucleotide sequence even within each type [1], [2], [3]. These introns are also mobile genetic elements, capable of movement into other genes and, in many cases, they contain one or more genes that encode for proteins (e.g. reverse transcriptase) that enable this mobility [4], [5]. Both types of introns have a wide phylogenetic distribution (Table 1), being found in bacteria and the organelles of plants, fungi, protists and animals [3], [6], [7], [8]. Interestingly, group II introns, although completely absent in nuclear eukaryotic genomes, are believed to be the ancestors of spliceosomal introns and therefore have played a central role in eukaryotic genome evolution [9]. In contrast, group I introns are found in phage, viruses and nuclear genomes of fungi and protists [3], [10], [11], [12], [13].

**Table 1:** Key characteristics of group I and group II self-splicing introns.

	Group I introns	Group II introns	*Nephtys* Intron
Distribution	Bacteria, virus, phage and organelles of plants, fungi, and some animals (sponges and cnidarians), nuclear genomes of protists and fungi [8], [13]	Bacteria, organelles of plants, fungi, protists, and animals (*Trichoplax* and *Nephtys*) [6], [7]	*Nephtys* mitochondria
Size (depends on the presence/absence of ORFs)	0.25–3.0 kb [3], [41]	0.38–3.4 kb[6]	1.8 kb
Secondary structure	Conserved within this group	Conserved within this group	Conserved with other group II introns
Splicing	Self-splicing without lariat formation	Self-splicing with lariat formation	Potential for lariat formation
ORFS	Homing endonucleases [3], [5]	Reverse transcriptase, maturase, DNA binding, and/or endonuclease domains [25]	Reverse Transcriptase and partial maturase

The very rare presence of introns in animal mtDNAs challenges current views on organelle evolution. With more than 900 complete animal mtDNA sequences available, only some members of the basal groups, sponges and cnidarians (which have group I introns) and the placozoan *Trichoplax adhaerens* (which has multiple introns, including one of group II), have been found to possess introns, and all appear to have been acquired secondarily [7], [10], [12]. Although mitochondrial genomes of protists, fungi, and plants display wide variation in size, structure, and gene content, undergo high rates of recombination, and often contain large amounts of non-coding sequence and both types of self-splicing introns [14], [15], [16], those of animals have become practically evolutionarily static, with almost all being small, compact, circular molecules with the same 37 genes and lacking introns, all but small tracts of non-coding sequence, and all or nearly all recombination [17]. Understanding the forces responsible for these restricted set of changes in animal mtDNAs, in contrast to those of fungi and plants, has been the subject of much study and debate among evolutionary biologists [18].

## Results

**Figure 1:**
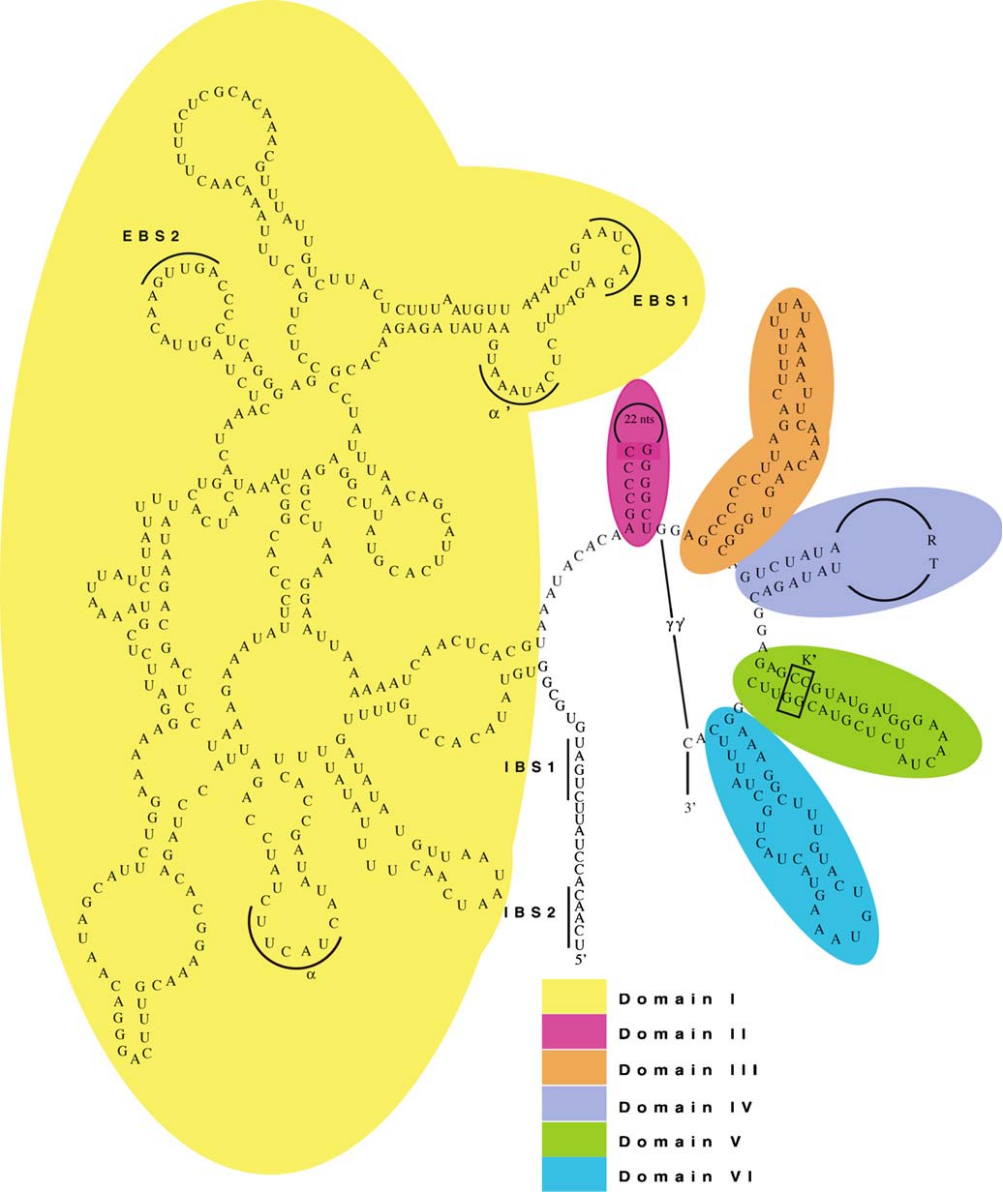
Predicted secondary structure of the *Nephtys* sp. group II intron. Potentially conserved secondary structure consisting of a central core from which radiate six domains (I–VI). The RT and partial maturase ORF are encoded within domain IV. EBS and IBS indicate sites where interaction between the intron and exon (respectively) occurs when splicing. Greek symbols designate sequence sites potentially involved in tertiary structure.

We have determined the complete sequence of the mitochondrial genome of *Nephtys* sp., a carnivorous polychaete inhabiting the intertidal and subtidal zones. This genome is typical of animal mtDNAs in possessing 37 genes on a single circular molecule with few and short non-coding regions [17]. However, contrary to all expectations, the protein coding gene *cox1* contains a group II intron (Fig. 1). We confirmed that the intron is a part of the mtDNA rather than a nuclear pseudogene by using polymerase chain reactions (PCR) to amplify the entire mtDNA in two overlapping pieces using inverted primers that anneal within the intron (Table 2). We verified that this intron is, in fact, removed from the mRNA by cloning and sequencing cDNA made from the transcripts of the *cox1* gene. We identified it as a group II self-splicing intron by a detailed examination of its sequence and potential secondary structure that revealed these diagnostic features: (1) conserved GUGYG and AY nucleotides at the 5′ and 3′ intron boundaries, respectively; (2) conserved sequence of domain V, which is the catalytic core of the intron's ribozymic activity; (3) presence of an ORF for a contiguous reverse transcriptase (RT) gene and a partial maturase gene; (4) potential secondary structure with six helical domains radiating from a central core consistent with the highly conserved secondary structure of group II introns (Fig. 1) [2], [6], [19], [20]. This is the first case of any intron found in the mtDNA of any bilaterian animal (Table 1).

**Table 2:** Primers used for completion of *Nephtys*' mtDNA amplification.

Universal primers [29]	LCO1490	5′- GGTCAACAAATCATAAAGATATTGG-3′
	HCO2198	5′- TAAACTTCAGGGTGACCAAAAAATCA-3′
Specific primers	Neph-cx1F	5′- TGGCCAACCAGGCGCACTTTTAGG-3′
	Neph-cx1R	5′- TGTAGCAACTACAGATCGTTGGG-3′
	Intron-F	5′- TCAGGGACAATAGCATTCTGG-3′
	Intron-R	5′- AACCGTACGAGATAGTTTCCC-3′
	A.186. 4C-cb-F-216	5′- AAGATTGCTTTTCACACATATTACACCG-3′
	A.186. 4C-cb-R-306	5′- GTTGTCAGGATCGCCTAAAGAAGTAGG-3′

**Figure 2:**
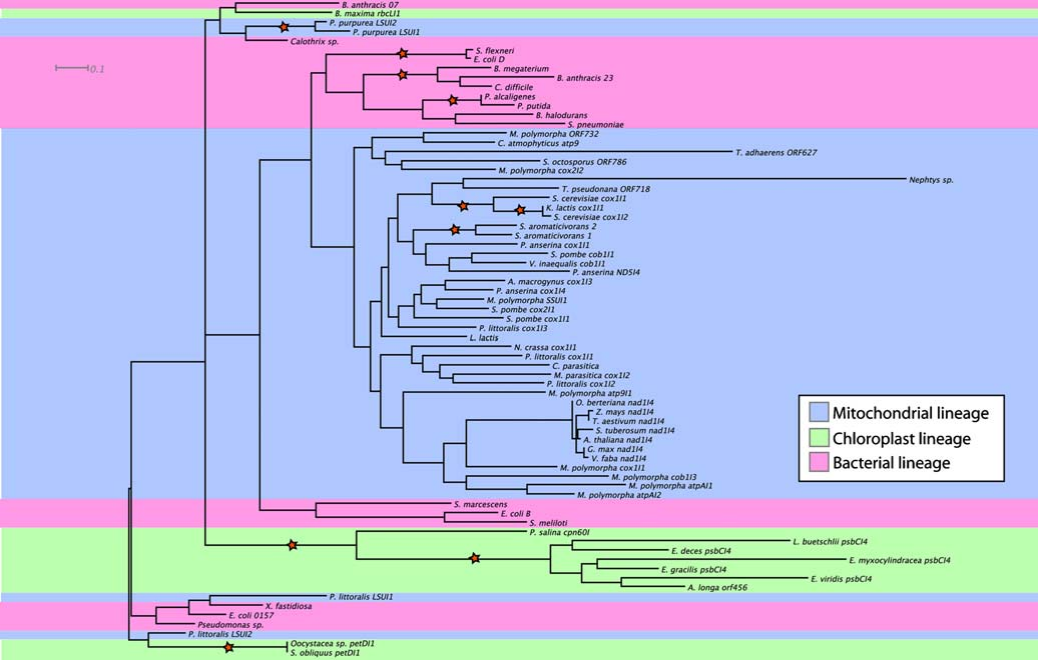
Phylogenetic analysis of 71 group II intron ORFs. A maximum likelihood analysis of the amino acid sequence for 71 ORFs suggests the *cox1* ORF718 of the marine centric diatom *Thalassiosira pseudonana* as sister to the *Nephtys*'s ORF. Red stars indicate a bootstrap support ≥90. Names of taxa are indicated by the capital letter of the genus name, followed by species name and when applicable the intron location (specified in table 3).

Attempting to identify the evolutionary origin of the intron, we incorporated the inferred amino acid sequence of the intron's ORF (RT and partial maturase) into the alignment of Zimmerly et al. [19], which contains ORFs from other group II introns from bacterial and organellar genomes (both mitochondrial and chloroplast) from plants, fungi and protists (Table 3). In addition we have included the three most similar sequences in a search (using BLAST) of GenBank to the intron's ORF of *Nephtys* and of *Trichoplax adhaerens* in the alignment. A maximum likelihood phylogenetic analysis suggests that *Nephtys*'s ORF is sister to the *cox1* ORF718 of the marine centric diatom *Thalassiosira pseudonana* among those RT sequences available for comparison (Fig. 2). However, broader taxon sampling of group II introns is needed to reliably infer their evolutionary history.

**Table 3:** Mitochondrial, chloroplast and bacterial group II introns included in the phylogenetic analysis (modified from Zimmerly et al. ).

Organism class	Species name	Host gene	Intron
Placozoans	Trichoplax adhaerens	*cox1*	
Bilaterians	*Nephtys* sp.	*cox1*	
Yeast	*Kluyveromyces lactis*	*cox1*	I1
	*Saccharomyces cerevesiae*	*cox1*	I1
	*Saccharomyces cerevesiae*	*cox1*	I2
	*Schizosaccharomyces pombe*	*cob1*	I1
	*Schizosaccharomyces pombe*	*cox1*	I1
	*Schizosaccharomyces pombe*	*cox2*	I1
	*Schizosaccharomyces octosporus*	*cox1*	I4
Other Fungi	*Allomyces macrogynus*	*cox1*	I3
	*Cryphonectia parasitica*	No data	No data
	*Neurospora crassa*	*cox1*	I1
	*Podospora anserina*	*cox1*	I1
	*Podospora anserina*	*cox1*	I4
	*Podospora anserina*	*nad5*	I4
	*Venturia inaequalis*	*cob1*	I1
Green algae	*Bryopsis maxima*	*rbcL*	I1
	Chlorokybus atmophyticus	*atp9*	
	*Oocystacea* sp.	*petD*	I1
	Scenedesmus obliquus	*petD*	I1
Liverworts	*Marchantia polymorpha*	*atpA*	I1
	*Marchantia polymorpha*	*atpA*	I2
	*Marchantia polymorpha*	*atp9*	I1
	*Marchantia polymorpha*	*cox1*	I1
	*Marchantia polymorpha*	*cox1*	I2
	*Marchantia polymorpha*	*cob1*	I3
	*Marchantia polymorpha*	*cox2*	I2
	*Marchantia polymorpha*	SSU rDNA	I1
	*Marchantia polymorpha*	Free standing	Orf732
Other green plants	*Arabidopsis thaliana*	*nad1*	I4
	*Glycine max*	*nad1*	I4
	*Oenothera berteriana*	*nad1*	I4
	*Solanum tuberosum*	*nad1*	I4
	*Triticum aestivum*	*nad1*	I4
	*Vicia faba*	*nad1*	I4
	*Zea mays*	*nad1*	I4
Red algae	*Porphyra purpurea*	LSU rDNA	I1
	*Porphyra purpurea*	LSU rDNA	I2
Brown algae	*Pylaiella littoralis*	LSU rDNA	I1
	*Pylaiella littoralis*	LSU rDNA	I2
	*Pylaiella littoralis*	*cox1*	I1
	*Pylaiella littoralis*	*cox1*	I2
	*Pylaiella littoralis*	*cox1*	I3
Stramenopile	Thalassiosira pseudonana	*cox1*	
Cryptomonad	*Pyrenomonas salina*	*cpn60*	I1
Euglenoids	*Astasia longa*	Free standing	Orf456
	*Euglena deces*	*psbC*	I4
	*Euglena gracilis*	*psbC*	I4
	*Euglena myxocylindracea*	*psbC*	I4
	*Euglena viridis*	*psbC*	I4
	*Lepocinclis buetschlii*	*psbC*	I4
Bacteria	*Bacillus anthracis*	None	
	*Bacillus anthracis*	PX01-08/PX01-06	
	*Bacillus halodurans*	PX01-24/ORFX	
	*Bacillus megaterium*	None	
	*Calothrix sp.*	ORF1	
	*Clostridium difficile*	ORF14	
	*Escherichia coli*	None	
	*Escherichia coli*	ORFH	
	*Escherichia coli*	IS629	
	*Lactococcus lactis*	Relaxase	
	*Pseudomonas alcaligenes*	No data	
	*Pseudomonas putida*	No data	
	*Pseudomonas* sp.	None	
	*Serratia marcescens*	None	
	*Shigella flexneri*	IS629-likeORF	
	*Sinorhizobium meliloti*	ORF B	
	*Sphingomonas aromaticivorans*	Replication primase	
	*Sphingomonas aromaticivorans*	ORF392/ORF416	
	*Streptococcus pneumoniae*	None	
	*Xilella fastidiosa*	DNA Methyltransferase	

## Discussion

The amino acid sequences of the intronic ORFs of *Nephtys* sp. and *T. adhaerens* (the only other animal shown to have a group II intron) have only 29% identity, indicating that these introns diverged long ago, presumably long-predating the divergence of these animal groups. Although both the *T*. *adhaerens* and *Nephtys* ORFs are found in the *cox1* mitochondrial gene, their positions differ by 108 nucleotides. For these reasons it seems very likely that this intron has integrated into these two mtDNAs in separate events, particularly because the alternative would require the hypothesis of many parallel losses in related lineages.

Thus, this group II intron is most likely the result of recent horizontal gene transfer, presumably from a bacterial or a viral intermediary. This would require that the transferred genetic material was specifically sequestered by the germline in order to be inherited. Interestingly, some bacterial lineages (i.e. *Wolbachia*) invade the female reproductive tissues of their host (i.e. *Drosophila*) and live intracellularly inside of the eggs, leading to inheritance of the microbial population in subsequent generations [21]. Furthermore, horizontal gene transfer from these endosymbionts to the host has been documented [22]. In the case of annelids with high regenerative abilities such as *Nephtys* [23], [24], it is also conceivable that the initial horizontal transfer event occurred in tissue that later re-differentiated during regeneration. Because these introns are highly mobile and move throughout populations by both horizontal transfer and vertical inheritance [2], [16], [25], the mitochondrial host could have acquired the intron from a bacterial endosymbiont. However, whether *Nephtys* sp. harbors endosymbionts is yet unknown.

**Figure 3:**
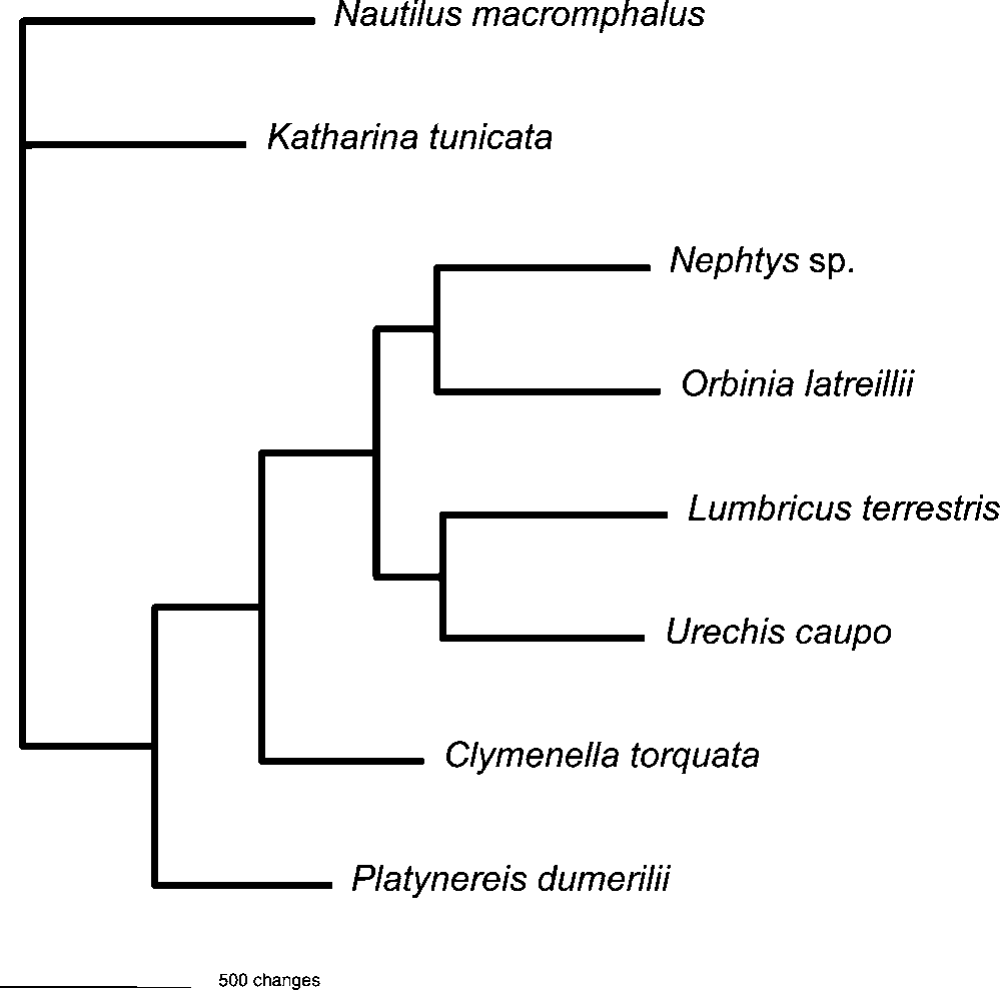
Maximum likelihood analysis of the protein coding genes. The maximum likelihood analysis of the mitochondrial protein coding genes of six annelids shows that branch lengths among them are similar, suggesting that *Nephtys* does not have an obviously slower rate that might create a propensity for harboring introns.

It may be that this intron is present only because it was recently acquired and insufficient time has passed for it to be lost. Nonetheless, it is tempting to speculate on whether there are properties of this mitochondrial genome that differ from those of most animals that have allowed it to escape from the presumed selection for small size to ensure rapid replication. Lynch and colleagues [18], [26] have advanced a theory to explain the opposite trends in the evolution of plant and animal mtDNAs that links rate of nucleotide substitution (much lower for plants) with the propensity to accumulate non-coding DNA (much higher in plants). Supporting this hypothesis, it has already been noted that, in contrast to most animals, cnidarians have a very slow rate of mitochondrial sequence change that may account for their adoption of introns [27], [28]. However, this does not appear to be consistent in the case of *Nephtys* sp., since a maximum likelihood analysis including all annelids for which the complete mtDNA sequence is available shows no great differences in branch lengths among them, even though only *Nephtys* sp. has acquired an intron (Fig. 3).

Further study of mtDNAs of annelids, as well as the other groups that have acquired introns (i.e. sponges, cnidarians and placozoans) may illuminate the extent and patterns of intron gain as well as provide further genome-level data for better understanding the forces shaping mitochondrial genome evolution.

## Materials and Methods

### Nucleic acid extraction and sequencing

Total genomic DNA was extracted from frozen tissue using a Qiagen DNeasy kit according to supplier's instructions. The mtDNA was amplified by long PCR (using the Takara polymerase kit) in three overlapping pieces (∼8 kb each) using specific and universal primers (Table 2) [29]. Each amplification product was ethanol-precipitated with NaSO_4_, dried, and resuspended in 100 µl of water. This was sheared into ∼1.5 kb fragments using a Hydroshear device (GeneMachines), then the fragment ends were repaired using Klenow fragment and T4 polymerase. The product was size-selected using an agarose gel and ligated into pUC18 vector. These clones were introduced into *E. coli* cells by electroporation. This was plated and grown overnight at 37°. Colonies were picked and processed to generate reads from each end of randomly selected clones.

Total RNA was extracted from tissues samples using Quiazol reagent (Quiagen) according to the manufacturers instructions. cDNA was constructed from total RNA using the Superscript III First-Strand Synthesis System for RT-PCR (Invitrogen) following the manufacturers instructions. In order to verify that the presumed intron was removed from the transcript, a portion of the *cox1* mRNA was amplified by PCR using primers matching the mtDNA sequence, and this product was isolated, cloned, and sequenced as above.

### Sequence annotation, alignment and phylogenetic analyses

Phred and Phrap were used to call bases and produce an alignment (∼10×) and Consed was used for manual verification of quality [30], [31], [32]. The mtDNA was annotated using DOGMA [33] and MacVector (Accelrys). The intron secondary structure was folded initially with Mfold [34] followed by hand editing.

In order to evaluate the evolutionary history of the introns themselves, the intronic ORFs within *Nephtys* sp. (Accession number EU293739), *Trichoplax adhaerens* [NC_008151], *Thalassiosira pseudonana* (ORF718) [YP_316587.1], *Schizosaccharomyces octosporus* (ORF786) [NP_700371.1] and *Chlorokybus atmophyticus* (ORF845) [NC_009630.1] were incorporated within the Zimmerly et al. [19] alignment using DIALIGN [35] (alignment accession number ALIGN_001217). Gblocks was used to determine the amino acid positions included in the phylogenetic analyses [36]. We performed a maximum likelihood (ML) analysis following the JTT model for amino acid substitution and executed bootstrap resampling to evaluate branch support in RAxML [37]. Percentage identity between *Nephtys*'s ORF and *T. adhaerens* was calculated using the amino acid sequence included in the analysis with the program WU-blastp (http://www.proweb.org/proweb/Tools/WU-blast.html).

In order to evaluate the rates of change for *Nephtys* sp. and related mtDNAs, the protein coding genes of six annelids (*Lumbricus terrestris, Nephtys* sp., *Clymenella torquata, Platynereis dumerilii, Orbinia latreillii,* and *Urechis caupo*) and two mollusks (*Nautilus macromphalus* and *Katharina tunicata*) were aligned with CLUSTAL X [38]. Gblocks [36] was used to determine the positions included in the analyses. Modeltest determined the ML model (TVM+G) that best fit the data. We built the tree (Fig. 3) using the settings given by Modeltest [39] (Lset: Base = (0.3156 0.2146 0.1402) Nst = 6 Rmat = (1.1922 12.4843 2.0012 5.9256 12.4843) Rates = gamma Shape = 0.2388 Pinvar = 0) and 1000 bootstrap replicates in PAUP [40].
